# Developing a Chatbot to Support Individuals With Neurodevelopmental Disorders: Tutorial

**DOI:** 10.2196/50182

**Published:** 2024-06-18

**Authors:** Ashwani Singla, Ritvik Khanna, Manpreet Kaur, Karen Kelm, Osmar Zaiane, Cory Scott Rosenfelt, Truong An Bui, Navid Rezaei, David Nicholas, Marek Z Reformat, Annette Majnemer, Tatiana Ogourtsova, Francois Bolduc

**Affiliations:** 1 Department of Pediatrics University of Alberta Edmonton, AB Canada; 2 School of Physical & Occupational Therapy McGill University Montreal, QC Canada

**Keywords:** chatbot, user interface, knowledge graph, neurodevelopmental disability, autism, intellectual disability, attention-deficit/hyperactivity disorder

## Abstract

Families of individuals with neurodevelopmental disabilities or differences (NDDs) often struggle to find reliable health information on the web. NDDs encompass various conditions affecting up to 14% of children in high-income countries, and most individuals present with complex phenotypes and related conditions. It is challenging for their families to develop literacy solely by searching information on the internet. While in-person coaching can enhance care, it is only available to a minority of those with NDDs. Chatbots, or computer programs that simulate conversation, have emerged in the commercial sector as useful tools for answering questions, but their use in health care remains limited. To address this challenge, the researchers developed a chatbot named CAMI (Coaching Assistant for Medical/Health Information) that can provide information about trusted resources covering core knowledge and services relevant to families of individuals with NDDs. The chatbot was developed, in collaboration with individuals with lived experience, to provide information about trusted resources covering core knowledge and services that may be of interest. The developers used the Django framework (Django Software Foundation) for the development and used a knowledge graph to depict the key entities in NDDs and their relationships to allow the chatbot to suggest web resources that may be related to the user queries. To identify NDD domain–specific entities from user input, a combination of standard sources (the Unified Medical Language System) and other entities were used which were identified by health professionals as well as collaborators. Although most entities were identified in the text, some were not captured in the system and therefore went undetected. Nonetheless, the chatbot was able to provide resources addressing most user queries related to NDDs. The researchers found that enriching the vocabulary with synonyms and lay language terms for specific subdomains enhanced entity detection. By using a data set of numerous individuals with NDDs, the researchers developed a knowledge graph that established meaningful connections between entities, allowing the chatbot to present related symptoms, diagnoses, and resources. To the researchers’ knowledge, CAMI is the first chatbot to provide resources related to NDDs. Our work highlighted the importance of engaging end users to supplement standard generic ontologies to named entities for language recognition. It also demonstrates that complex medical and health-related information can be integrated using knowledge graphs and leveraging existing large datasets. This has multiple implications: generalizability to other health domains as well as reducing the need for experts and optimizing their input while keeping health care professionals in the loop. The researchers' work also shows how health and computer science domains need to collaborate to achieve the granularity needed to make chatbots truly useful and impactful.

## Introduction

Knowledge exchange in the medical domain presents multiple challenges, including accessibility, readability, and accuracy. Chatbots, or computer programs that simulate conversations, can help answer users’ or caregivers’ questions [1,2]. Moreover, a chatbot offers several advantageous features for medical needs: flexibility (service providers available 24/7 and from any location [3]), speed (rapid delivery of a large number of resources), privacy (confidential access for users), engagement (an appealing user interface that fosters interaction), and trustworthiness (information developed by professionals, ensuring reliability). Chatbots have already been developed for diagnosing heart disease [4,5], providing counseling for mental health [6], improving patient monitoring and medical services [7], and preventing eating disorders [8]. Some chatbots have integrated coaching elements to support youth with weight management and prediabetes symptoms [9,10], young adults with depression and anxiety [3,11], people with obesity and emotional eating issues, adults wishing to improve wellness [4,5], and young adults with a high level of stress [6]. User trust remains a key challenge faced by medical and health-related chatbots [12].

Chatbots powered by advances in natural language processing (NLP) such as large language models (LLMs; eg, ChatGPT [7]) have shown how chatbots can revolutionize the way information is shared and accessed.

Nonetheless, developing a chatbot for the medical domain is challenging, especially when targeting complex medical conditions such as neurodevelopmental disorders (NDDs). NDDs represent a diverse group of conditions affecting development, including conditions such as attention-deficit/hyperactivity disorder (ADHD), intellectual disability, autism spectrum disorder, cerebral palsy, and learning difficulty. Together, NDDs affect up to 14% of children [8] and have major implications not only for the individuals themselves but also for their families and society [13]. It is increasingly recognized that individuals with 1 NDD diagnosis (eg, autism spectrum disorder) often also present with features of ADHD or learning difficulty. Moreover, several associated conditions (often referred to as comorbidities) [14,15] can also significantly affect the clinical presentation of individuals with NDDs and their needs in terms of health, social participation, and education; for instance, sleep disorders, gastrointestinal symptoms, anxiety, depression, or even seizures are more commonly found in individuals with NDDs than in the general population. NDDs are also increasingly linked to genes. While this has paved the way for personalized medicine, it has also led to silos where parents have access to information only through associations created for their genes of interest, resulting in very limited information in terms of management for many rare conditions. Furthermore, NDDs are chronic conditions with changing manifestations and needs over the course of an individual’s lifespan.

While LLM technology is evolving quickly, here, we discuss key steps to be considered when developing a chatbot for the medical domain and present how our team applied these steps to our use case of developing a chatbot for medical information related to NDDs named CAMI (Coaching Assistant for Medical/Health Information).

## Methods

### Consulting End Users About Their Needs to Target the Needs of Potential Users

It is important to consult directly with individuals with lived experience. While this is now common practice in industry, it remains challenging to reach out to individuals with medical issues.

#### Step 1: We Recommend Developing an Advisory Group That Includes Representative Individuals

In our case, we developed a national advisory group that included 9 caregivers of individuals with NDDs (we advertised for the advisory group position through associations and partners involved in NDD research in Canada). The caregivers were predominantly female (8/9, 89%), and their ages ranged from 32 to 51 years. In terms of marital status, of the 9 participants, 7 (78%) were married or had common law spouses, and 2 (22%) were divorced or separated. They had diverse levels of education: bachelor’s degree (7/9, 78%), master’s degree (1/9, 11%), and PhD (1/9, 11%). Of the 9 participants, 6 (67%) were employed full time, while 3 (33%) worked part time. The participants had various occupations: special education teacher (1/9, 11%), planning and programming officer (1/9, 11%), senior IT consultant (1/9, 11%), community development coordinator (1/9, 11%), academic professor (2/9, 11%), research assistant (1/9, 11%), psychologist (1/9, 11%), and manufacturer (1/9, 11%). Of the 9 individuals, 8 (89%) were White, and 1 (11%) identified as a First Nations person. All participants were biological parents.

The advisory group will not only provide direct feedback on the project but will also, through their personal and professional networks, recruit participants for testing the ideas or chatbot prototype.

#### Step 2: Ethical Considerations

It is important to prepare in advance a complete set of questions to be used in semistructured interviews with individuals with lived experience. The advisory group can provide key insights into the development of the consent forms, study protocol, and interview material. For developers from industry, it is key to collaborate with clinicians, association leaders, or other allied health professionals who are not only able to provide feedback but can also help engage participants in the project.

In our case, the project was approved by the research ethics board at the University of Alberta (Pro00081113). Informed consent was obtained from participants in interviews. The participants were compensated for their work as per ethics regulation.

#### Step 3: Consultations

We used semistructured interviews (Multimedia Appendix 1) adapted from user interface evaluation resources [12,16-18] to ask advisory group members about their current needs, their use of web-based platforms to gather information, and the current barriers to access to information. All interviews were video recorded after receiving consent from the participants.

Our interviews allowed us to identify overlapping themes using thematic analysis [19]. We were able to identify the patterns and themes among different user groups and build a plan on how to represent the data. The advisory group members (who were individuals with lived experience) suggested that the chatbot should provide rapid access to information:

I like the way that the resources just pop up in the side window as the user is answering the questions.

You could be asking me questions all day long but I’m not sure how close it can come to identifying the biggest stressors and challenges for our family. According to those responses they could target the info resources accordingly. If the chatbot doesn’t get to the bottom of our challenges fast enough, as a parent, I would likely find myself turning to Google more quickly to find what I’m looking for.

#### Step 4: Knowledge Mapping

It is important to identify the potential differences in the conceptual framework associated with medical conditions. These differences in mental representations, known as mental models, can lead to blind spots that may prevent the chatbot from being widely useful [20].

### Developing a Database of Resources for the Chatbot

#### Step 1: Gathering Information

Providing trusted, exhaustive, and actionable information is key for a chatbot meant to provide medical information. It is important to partner with individuals with lived experience, associations (patients, parents, and professionals) relevant to the condition, and health authorities to obtain such information. The information about the relevant web pages, books, or other formats needs to be stored centrally and visible to others so that it can be peer reviewed. This can be done using shared sheets or websites.

In our case, we developed a database of NDD resources by leveraging existing databases (Alberta Children’s Hospital, AIDE Canada, and InformAlberta [21,22]) as well as by developing a nationwide consultation with individuals with lived experience from across Canada who submitted 1422 resources. AIDE Canada and InformAlberta shared their resource database with the research team, which has been incorporated in the main CAMI database. The same data set or a superset of the data is used on the general websites of the aforementioned organizations. The individuals with lived experience used Google Sheets to add the known or found resources with the appropriate data labels. These data included the web page URL, type of resource, language, age group, location, eligibility criteria, and some important keywords. All resources were collected and annotated manually by group members. Submitted resources covered several topics such as health, education, and support programs and were grouped under the categories *core knowledge*, educational, *financial support*, and *services*.

**Figure 1:**
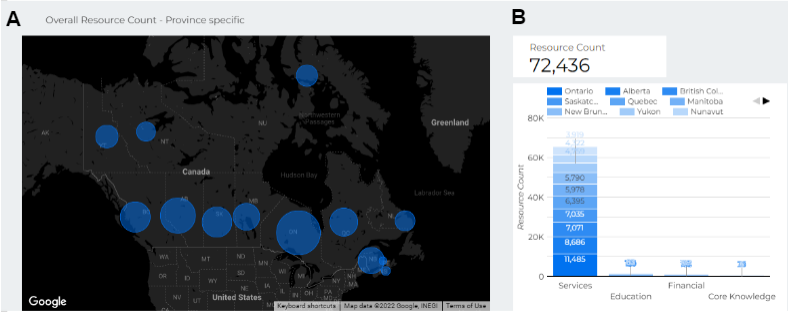
Representation of the geographic distribution of neurodevelopmental difference resources. (A) The number of resources per province and territory is represented with a circle (blue) on the map of Canada. (B) The distribution of resources by type of resource (services, core knowledge, financial support, or education) is shown.

A key feature brought up by the families of individuals we consulted was the need for an in-depth, trusted, and diverse set of resources. Therefore, we collaborated with several organizations involved in providing NDD treatment as well as individuals with lived experience. We encountered challenges in obtaining resources for diverse NDD subtypes and covering all regions of Canada at the start because we connected only with organizations that tend to have better coverage in urban areas. We found that forming a network of individuals with lived experience was very helpful and allowed us to achieve broader coverage in identifying resources (Figure 1; Textbox 1).

**Number of web resources per province and territory in the CAMI (Coaching Assistant for Medical/Health Information) database. The textbox shows the number of resources available in each province in descending order. The resource count includes all categories available for each province.**

**Province and number of resources**

- Ontario: 11,819
- Alberta: 8986
- British Columbia: 7330
- Saskatchewan: 7285
- Quebec: 6640
- Manitoba: 6218
- New Brunswick: 6095
- Yukon: 4951
- Nunavut: 4508
- Newfoundland and Labrador: 4085

#### Step 2: Data Annotation

It is important to annotate the resources included in the data set in a meaningful way so that the information can be retrieved in response to related queries by future chatbot users. Again, discussions with potential users are key in capturing topics of interest and making sure that they are properly covered in the database.

**Figure 2:**
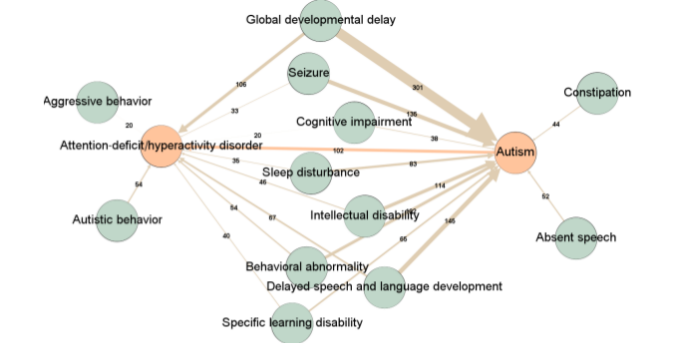
Knowledge graph of co-occurring entities in neurodevelopmental disabilities or differences. Co-occurring phenotypes for autism and attention-deficit/hyperactivity disorder in the Deciphering Developmental Disorders (DDD) database are represented as nodes. Arrows or connections between nodes represent the is_associated_with relationship. Numerical values represent the weight of the connections, which refers to the number of co-occurrences, and the thickness of the arrow is proportional to this weight. The Deciphering Developmental Disorders database contains 715 and 280 individuals with autism and attention-deficit/hyperactivity disorder, respectively.

We developed an automated annotation tool [23] using an NLP pipeline that uses a combination of named entity recognition, topic modeling, and text classification model to annotate the resources. All resource annotations along with their weight were stored in the Neo4j graph database (Neo4j, Inc) in the form of a weighted knowledge graph as shown in Figure 2 in the paper authored by Costello et al [23]. Ordered weighted aggregation operators are used to rank the resources that will be printed out in response to a user query.

Alternatively, one could use LLMs to extract keywords from the individual web pages. This would include selecting a suitable prompt and providing the web page content extracted from the URL. By leveraging LLMs such as ChatGPT and Google Gemini, among others, the content can be analyzed to identify and filter out important keywords from the web page.

#### Step 3: Database Formatting

The information is stored in the database in a format that retains the dependencies and relations among different data. The data are divided into multiple tables or classes such that the data are modular and can be used independently.

We used the Neo4j database to store the database models, and the database includes several nodes, relationships, and properties. The web page content was initially stored in the MongoDB database (MongoDB, Inc) and was further annotated into multiple properties and relationships. These properties are *is_about*, *is_associated_with*, *is_located_in*, *occurred_together*, and so on. When querying the database, these properties and corresponding relationships are extracted to filter out the resources from the database and rank them in accordance with the defined parameters.

### Designing and Evaluating the Chatbot User Interface Design

#### Overview

A crucial aspect of the chatbot relates to its user interface, which requires it to be functional, easy to use, and attractive so that it appeals to users [6-8,13]. Of particular importance is the landing page because it represents the first impression of the application and should connect with the target audience [7] by reflecting the user’s [6] as well as the chatbot’s goals [14]. Important components of the page design include a logo, design, fonts, colors, and layout [15] (Multimedia Appendices 2-7).

#### Step 1: Consultation Regarding the Interface

To streamline the process of multiple iterations, mock-up representations of the chatbot can be used in interviews with individuals with lived experience. This is important to increase the cost-effectiveness and reduce the time required for coding each version. In our case, the mock-ups, or the design wireframes of the chatbot, were shared with the participants (individuals with lived experience) by research team members on a Zoom call (Zoom Video Communications, Inc), and their feedback was recorded on Google Docs during these calls. We usually included both content experts and computer scientists to uncover gaps. This cycle should be repeated until a consensus is reached among the target user base, and the design is finalized [24]. In the design process, we used Figma (Figma Inc) [25], a collaborative design tool that allows users to work on various designs while also allowing others to review them and provide comments. Optimal user interface design uses an agile methodology that includes iterative design, implementation, and testing [26].

**Figure 3:**
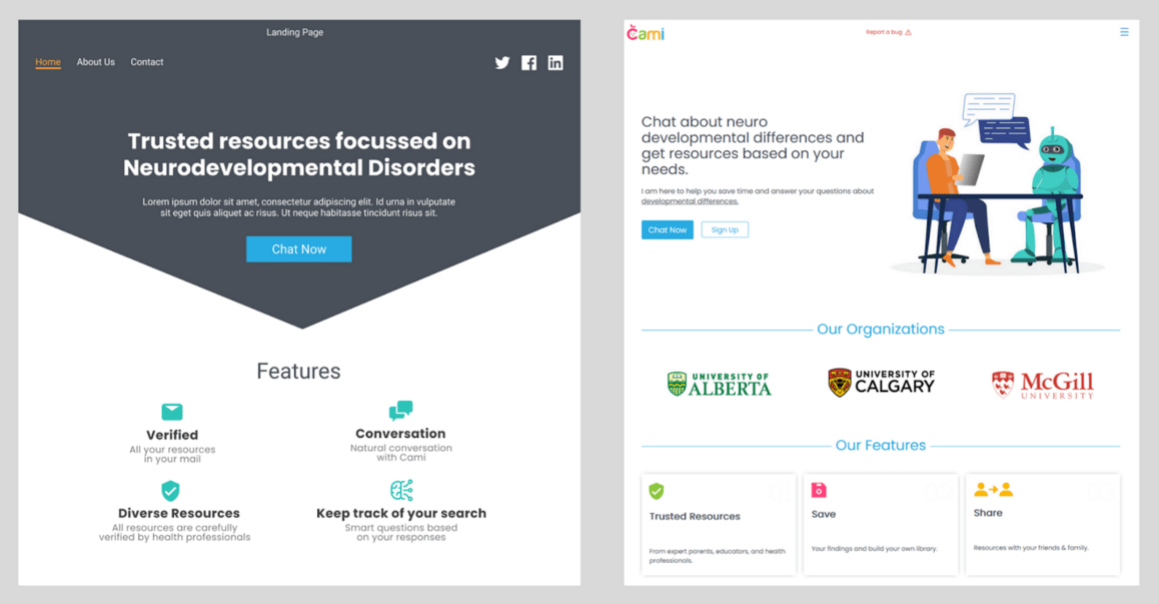
Evolution of the chatbot home page design. (A) The initial design of the home page by our team before consultation with caregivers, which displays text indicating that the resources are trusted and diverse but does not explain what the chatbot can do. (B) The updated home page, revised after several consultations with families. It has a neutral color scheme and a graphical visualization that represents a robot conversing with a human. Adding the names of partners or affiliated organizations on the home page establishes trust in the website and proves that the product was developed by reputed organizations.

We started with the home page of the chatbot (Figure 3). We found that families of individuals with NDDs favored a simple design with a clear indication of the purpose of the chatbot. We also included a tutorial video about the purpose and intended use of the chatbot. The families also recommended the inclusion of the names of the institutions involved in the development of the chatbot as a mark of trusted information.

The home page was designed using Gestalt principles [27], and all closely matching content was kept on the same page. During the initial interviews, participants indicated that the home page content was not self-explanatory, clear, and trustworthy. The feedback was considered and the required changes made to showcase the authenticity of the chatbot.

#### Step 2: Conversation Design

The conversation (or generic flow), which represents the exchange between the user and the chatbot, is a very important aspect in usability. Most chatbots will include an introductory text explaining the purpose of the chatbot. Subsequently, the conversation can be more or less prestructured.

An introductory video can help the user understand rapidly the aim of the chatbot as well as how best to ask questions. Importantly, the video needs to be short enough for the user to stay engaged with the chatbot.

**Figure 4:**
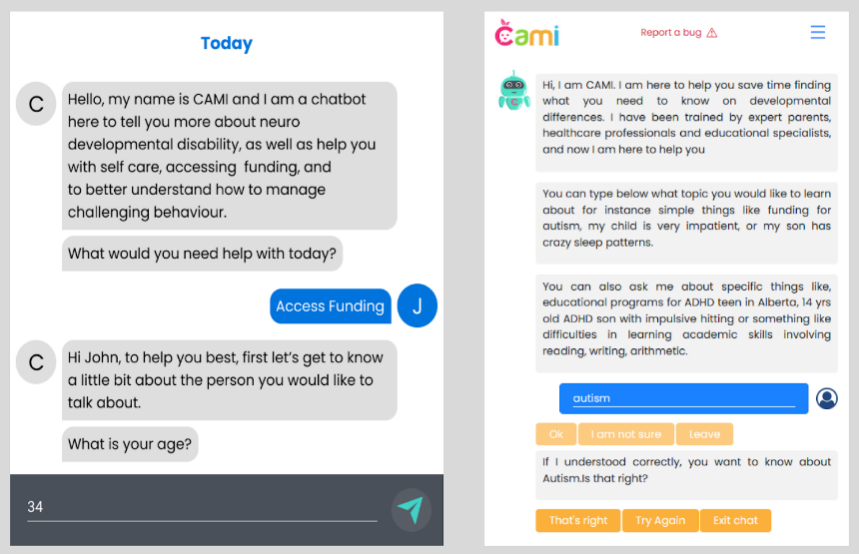
Representation of the conversation (chat). (A) In the initial design, before consultation with caregivers, the user had to always answer the question, and there was no way to correct the chatbot if it misunderstood. (B) The updated design, revised after several consultations with individuals with lived experience, is much more conversational and flexible for users, allowing them to skip, make corrections, or end the chat when they want to. The design also makes use of radio buttons (yellowish-orange) that can help the user to quickly acknowledge what the chatbot is saying or correct course if needed.

In our case, the conversation (chat) design went through several iterations based on input from caregivers of individuals with NDDs (Figure 4). In the initial design, the users were confused when the chatbot was not able to understand them. Either they had to start the chat again or follow along with the conversation. In the final design, users have the ability to inform the chatbot if its understanding is incorrect, and they can enter their query again. Users have more flexibility in responding to certain questions by clicking “I am not sure” if they are not comfortable with answering them. In certain cases, we also provided radio buttons based on participant feedback:

Instead of having the user type yes or no every time, could a selection not appear using radio buttons, one for yes and one for no? It would be easier for the user.

We developed a video explaining the aim and scope of the chatbot to avoid users asking questions outside of our domain of expertise. The video is available on the web [28].

#### Step 3: Output Representation

Another major focus was on how to present the results of the query.

**Figure 5:**
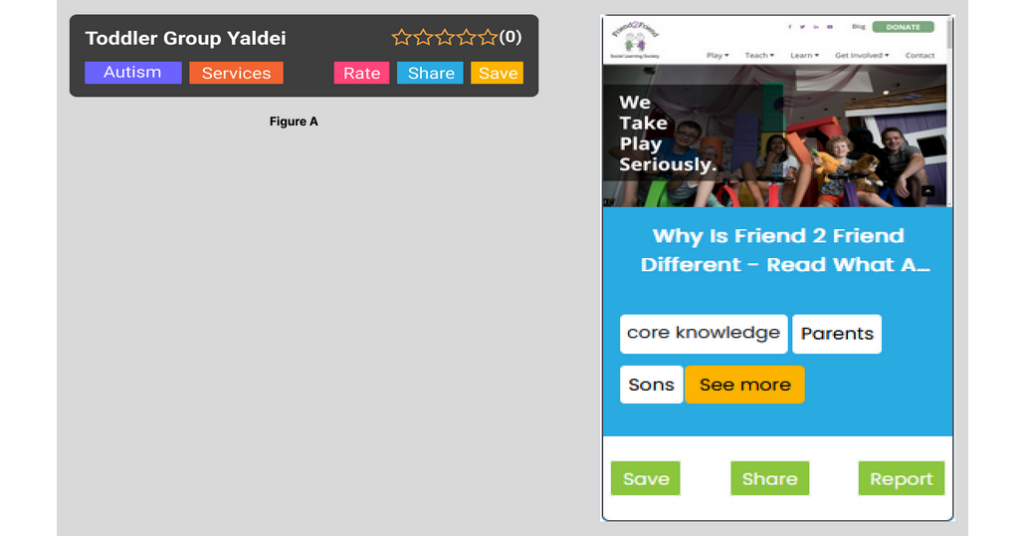
Representation of the resources identified by CAMI (Coaching Assistant for Medical/Health Information). (A) Early version of the resource display, before consultation with caregivers: only resource names and tags are shown to the user. (B) Updated resource card, revised after several consultations with families: each resource is represented with a static picture of the website, the website name, tags describing the types of resources (core knowledge and resources) as well as key concepts (age and location when appropriate). Radio buttons at the bottom allow users to either share the content with a friend (using SMS text messaging) or save a resource to their profile. They can also report any issue with the resource (eg, not trusted or inaccurate) to our team.

In our case, web resources identified by CAMI are presented to the user (Figure 5). This part underwent significant modification to provide information to users that would influence their decision to continue browsing or not. Users have major time constraints, and they would lose interest quickly if they are unclear about the quality of the resource. The resource card evolved from showing a title and rating to showing a static image of the website, the type of resource, tags describing the resource, and some key functions such as sharing or saving the resource (which was identified by users as key in being able to access the chatbot from multiple environments, eg, work, commute, and home). All static images are captured as screenshots and saved in the database. The families reported that in addition to being more appealing, the images would help to build trust and identify the authenticity of a site if they could see its home page. Moreover, users mentioned that they would be able to recognize and remember the site more effectively if it displayed an image. We also took advantage of the tags to allow the user to *converse* *with* or *direct* the chatbot dynamically. Indeed, when the user clicks on a radio button, say, “parent” or “autism,” the chatbot would respond by providing resources related to this tag. This not only allows the user to start with a query but also enables them to navigate to other topics of interest that they may not have initially considered.

**Figure 6:**
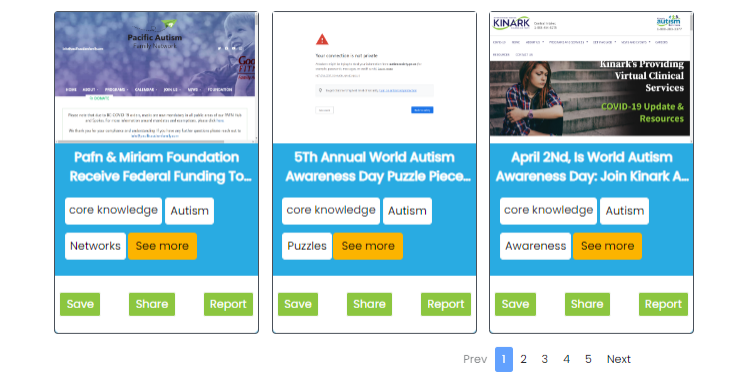
Resource presentation format. After receiving a query, the chatbot will provide a set of 3 resources to the user. The page numbers at the bottom indicate to the user that there are more resources available (when applicable). The user can click on Prev and Next to go to the other pages. They can also click on See More (yellowish-orange radio button) to check out more tags on the resources and decide whether they want to save, share, or report the resource.

Next, we investigated how many resources to display during a single interaction. The families’ feedback showed consensus at 3. Resources are presented in sets of 3 with page numbers at the bottom showing the user that there are more resources for them to look at in the future (Figure 6).

#### Step 4: User Engagement and Coaching Strategies

While websites have used several ways to encourage users to remain on their platform, it is important to consider the vulnerable nature of patients or their families as well as the potential detrimental effect of prolonged use. At the same time, encouraging the user to reconnect with the platform will allow repeated use and coaching.

Health coaching and care coordination [29,30] have been shown to improve health outcomes for individuals with NDDs, but they are not offered to most families because of cost, a lack of specialized health care professionals [31], access barriers due to geography, or a lack of integration into the health system (eg, insurance and social factors).

**Figure 7:**
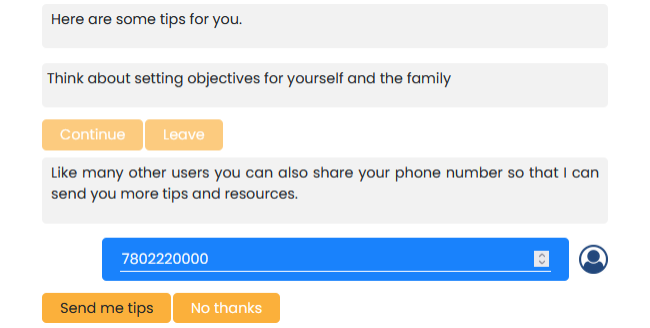
Tips provided by experts in neurodevelopmental disabilities or differences used by the chatbot. Conversation flow for sending users tips is shown, along with related information about their query. Users can also input their telephone number and receive other tips in the future.

In our case, we consulted with health experts in NDDs and identified key coaching aspects that could be provided to users. We also asked users whether they were interested in being sent tips or related resources via SMS text messaging or email (Figure 7).

### Optimizing User Input Understanding in Domain-Specific Terms

Understanding user input is an important factor in providing responses to user queries. NLP is a domain of artificial intelligence focused on developing computer programs that are able to *read* textual data, analyze the content, and extract meaningful information [32]. Advanced deep network NLP algorithms, such as named entity recognition and relation extraction, have been repurposed to identify diseases, symptoms, and drugs from user input [33-35]. Chatbots use NLP to process user text and respond to the text. While the underlying processes are different, chatbots aim to behave like people who listen and respond to their conversational partners. Day-to-day language is difficult for a computer to understand, but, using NLP, the computer is able to break up user text into a set of attribute-value pairs; for example, if the user types “I want to know if there are any services available for Global Developmental Delay in Edmonton,” the NLP pipeline would break up the text into subparts by extracting keywords: {*Services, Global Developmental Delay, Edmonton*}.

We leveraged an annotation NLP pipeline [23] to extract structured information from the user free-text query. Every query submitted by the user is divided into multiple medical-related domains such as Human Phenotype Ontology (HPO) for medical terms and Alliance of Information & Referral Systems (now known as Inform USA) for service-related terms, as well as symptom-specific terms (eg, challenging behavior), geographic location, and the age of the individual.

We found that formatting the query into multiple domains helped us identify the resources with more accuracy compared to performing a search using keywords. We experimented with certain queries from the test group and identified the related conditions for common queries. Textbox 2 shows the extraction of a single query to a list of multiple medical database keywords that relate to each other when filtering the resources.

In CAMI, certain keywords are hardcoded so that the recommendations can be domain-specific and adhere closely to user requirements. If keywords such as “knowledge” or “information” are included in the initial user query, the user will not be asked about their geographic location because location is not relevant; for instance, general knowledge regarding autism is likely consistent in Canada, the United States, or Germany, but services or policies will differ based on location. By contrast, if the keyword “services” is detected in the user query, and the location is not extracted from the query itself, the user will be prompted to provide their location (city or province) such that the recommended services can be actionable.

**Query analysis by domain. A query (eg, “My son is suffering from ADHD”) is analyzed by using multiple annotation sources: Human Phenotype Ontology (HPO) from the Deciphering Developmental Disorders (DDD) database, the Unified Medical Language System (UMLS), a service-based annotation using the Education Resources Information Center (ERIC) thesaurus, and a tool for annotating challenging behaviors at home. Ngrams represent topic keywords from topic modeling.**

**Term and value**

- HPO-DDD: “attention-deficit/hyperactivity disorder”
- UMLS: “mental suffering”
- ERICterm: “sons”
- cb_category: “behavioral concerns”
- Ngrams: “son, suffering, adhd”
- searchedCategory: “core knowledge”
- Location: “not found/not required”
- Age: “not found”

### Chatbot Framework Coding

There are several tools available to create chatbots with diverse functionalities and their use varies with each use case. Wit.ai [36], Rasa [37], and Dialogflow [38] are some of the popular frameworks used to design rule-based chatbots.

We used the Django library in Python to create the backend for CAMI. It follows the model-view-template framework and app-based architecture, in which all classes are called apps and can work independently as well as in conjunction with other apps in the system ecosystem [36]. Each app includes a model file, a views file, a test file, and an init file. The schema was defined for the database table in the *models.py* file, where the column name is declared with its definition, which includes data type, constraints, and default values. All changes in the database model generate a migration file, which is an auto-created SQL code that creates or updates the database’s internal schema [37,38]. View files include a set of functions with decorators that define the type of http request served [39]. Django (Django Software Foundation) was selected over comparable frameworks such as Flask because using Django’s authentication module streamlined the development process for registration and log-in functionalities through auto-encryption, password verification, and authorization [34,35].

**Figure 8:**
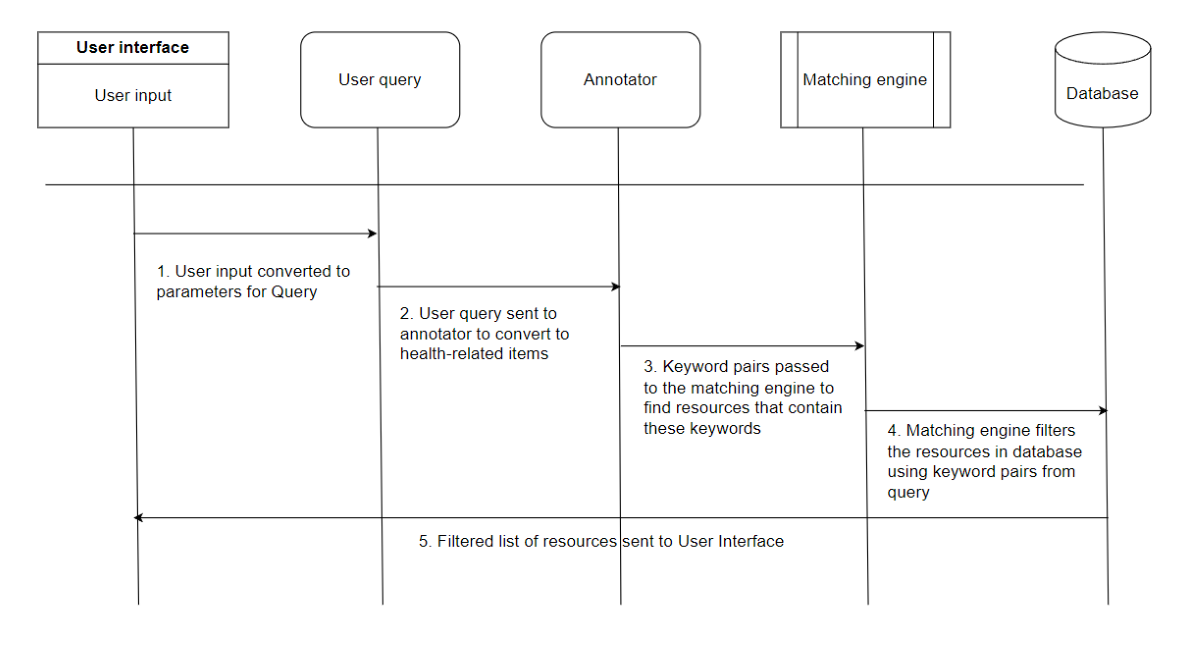
Chatbot workflow for query analysis. The inputs from the user interface are converted to the user query, which is passed to the annotator to procure health-related keyword pairs. The extracted data are sent to a matching engine for filtering. The matching engine checks the database to find the matching keywords and returns filtered resources.

Once the information is extracted from the user query, it is sent to the matching engine. The matching engine incorporate the logic to find the similarity between the given input data dictionary and the list of resources from the database. The resources are annotated in the same way as the query to maintain the uniformity between the query and the list of resources to be considered for matching after the resources are filtered with the type of resource requested (Figure 8).

**Figure 9:**
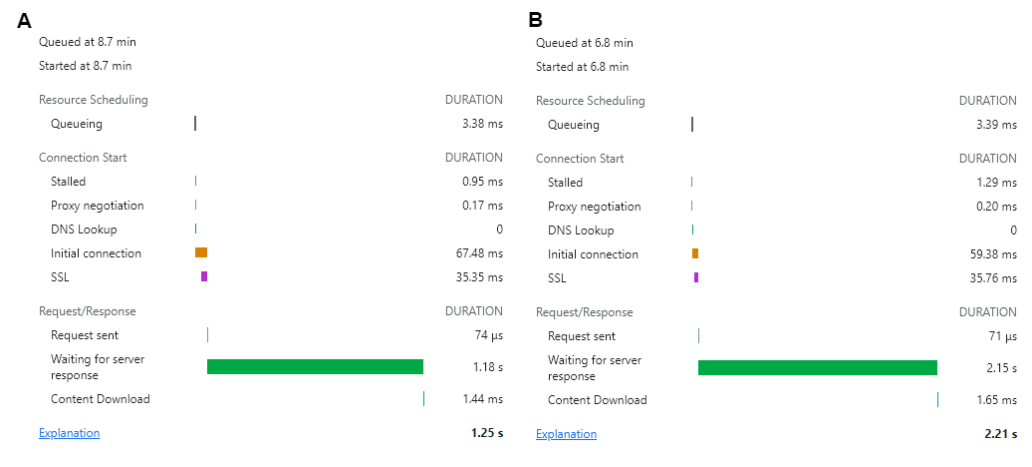
Query annotation time. Representation of examples of estimated time for the chatbot to analyze a user query. Two different queries are shown: (A) test 1 (“My child has sleep issues”) and (B) test 2 (“My child has sleep issues and he is suffering from ADHD and cries in the night”). (A) The application programming interface timing, which includes queuing, connectivity, and response return or throughput time, is displayed. (B) For a longer query (test 2), the same parameters as the test 1 query are displayed. SSL: secure sockets layer.

We optimized the system to obtain faster throughput to display the results faster. This was done by incrementing the heap size and page cache in the Neo4j configuration file. The average response time for query analysis is approximately 2 seconds, but this depends on the length of the sentence. For smaller sentence sizes, the output will be returned in a shorter time period (Figure 9).

**Figure 10:**
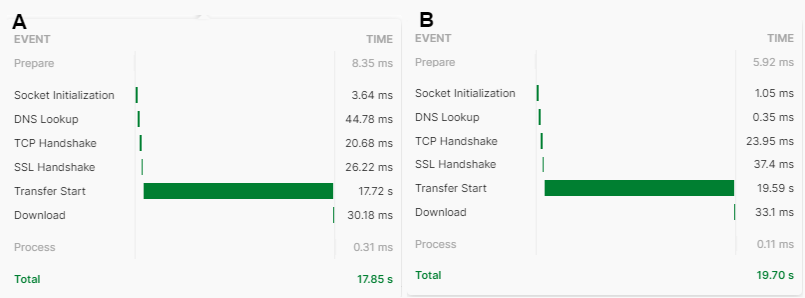
Chatbot recommendation time. “Socket Initialization” is an event that enables the client (browser and third-party application programming interface tool) to connect to the internet. “DNS [domain name system] Lookup” retrieves the IP address from the web by deciphering the domain name, and the “TCP [transmission control protocol] Handshake” establishes a reliable connection between client and server. The “SSL [secure sockets layer] Handshake” establishes a secure communication channel between client and server. “Transfer Start” refers to the retrieval of the complete data from the server. “Download” refers to the time required to obtain all the packets at the client destination. The response times for the recommendations for both (A) test 1 (“My child have sleep issues”) and (B) test 2 (“My child have sleep issues and he is suffering from ADHD and cries in the night”) are displayed.

Similarly, the time required to obtain the recommendations for the queries (Figure 9) changes with the size of the payload. The engine checks all web pages and ranks them on the basis of the topic, entity, and location (Figure 10).

In developing the chatbot, it is important to follow the CONSORT (Consolidated Standards of Reporting Trials) guidelines [39]. CONSORT guidelines are usually meant for reporting clinical trials, ensuring transparency and accuracy in the reporting of trial methods and results, but the same principles can be applied to software development as well. The guidelines include the following: (1) mention the names, credentials, and affiliations of the developers, sponsors, and owners; (2) describe the history and development process of the application and previous formative evaluations; (3) report revisions and updates; (4) provide information on quality assurance methods to ensure the accuracy and quality of the information provided; (5) ensure replicability by publishing the source code; (6) provide the URL of the application; however, because the intervention is likely to change or disappear over the years, make sure that the intervention is archived; and (7) describe how participants accessed the application, in what setting or context, and if they had to pay for access.

In our case, we applied the CONSORT guidelines as outlined in Textbox 3.

**Application of the CONSORT (Consolidated Standards of Reporting Trials) guidelines.**

**Adhering to the CONSORT guidelines**

1. We provided the following information about the developers and authors: Ashwani Singla (backend), Ritvik Khanna (front-end), Manpreet Kaur (NLP [natural language processing]), and Francois Bolduc (conceptual and content expert).
2. CAMI (Coaching Assistant for Medical/Health Information) went through multiple major changes throughout the project timeline as well as numerous ongoing minor changes that were tackled on the way to building the Epic (project milestone) or the goal of the system. All code changes are committed to the GitHub repository with separate branches for front-end, backend, and NLP. Any modification to the code will go to the respective branch, and the integration developer will perform integration testing to merge the code. All modifications to the chatbot are subject to feedback from the testing group and the principal investigators of the project.
3. CAMI went through 3 major releases and 403 commits on the GitHub repository [40]. The most recent stable version for the backend functionality was released on April 16, 2023, and some changes were made on the front-end progressively. Development was frozen for all versions during the testing phase so that the same system could be tested by multiple people. There were significant changes to version 3.0 with regard to using the knowledge graph to find the connection between the entities and share the information with users.
4. During testing, the chatbot's quality was evaluated based on 3 main components: user understanding of the chatbot's functionality, user satisfaction with the overall quality of resources, and user confidence in sharing information on the platform. These metrics helped the development team to enhance the quality of the resources by placing more relevant resources on the first page and others on the subsequent pages. Similarly, the question flow changes are a result of the confidence shown by users, which was evaluated based on the user being willing to answer at least 80% of the questions asked by the chatbot.
5. CAMI is deployed on the University of Alberta’s MedIT server with the URL [41]. The default page corresponds to the home page or landing page. The backend end points can be accessed using the same URL but with different subroutings.
6. The testing method varied based on the version of the chatbot. In the first and second test phases, the participants were on a video call on Zoom (Zoom Video Communications, Inc), and the screen view was shared with them to enable them to view the website. Participants were granted remote control access to the screen to test the system, although some participants guided the proctor to click certain options. In the third version of testing, the URL was shared with the participants so that they could use a web browser to test the system. Participants could report any bugs they found. Finally, the testing group completed a feedback form.
7. The chatbot is currently freely accessible.

### Establishing a Conversation Flow for the Chatbot

In the case of medical conditions, especially complex ones such as NDDs, patients present with variable or multiple issues that can all be of interest. Therefore, it is important to include a knowledge of such associated concepts to provide comprehensive information. In some cases, the core disease, say, autism or advanced stage cancer, may not be treatable, but associated conditions (eg, anxiety or constipation) may have interventions available to the patients. Concepts (or entities) and their relationships are often stored in knowledge graphs. A knowledge graph consists of a graphical and structured representation of information: terms or concepts can be stored as nodes that are connected to one another through edges that define different relationships [42]. Knowledge graphs are already being used to store information about disorders [8,13,14] and could therefore be leveraged by chatbots.

Using a knowledge graph in a health assistant or chatbot allows the question-answering system to provide (1) information regarding, for instance, disease symptoms [43,44]; (2) internet-based diagnosis and risk assessment [45]; (3) personal lifestyle interventions for serious conditions [46-48]; and (4) prediagnosis and triaging using the patient’s symptoms and medical history [49,50].

We developed a knowledge graph representing key entities observed in individuals with NDDs as well as their interrelation by leveraging the largest data set of individuals with NDDs, the Deciphering Developmental Disorders (DDD) database [51]. The DDD database includes phenotypic and genotypic information along with the ages of 13,424 patients with severe and undiagnosed NDDs [30]. The DDD database labels phenotypes using the HPO. The knowledge graph contains 4181 HPO phenotypes for which we calculated the co-occurrence counts among all patient profiles and stored them in the knowledge graph using the “is_associated_with” relationship. The knowledge graph contains 357,514 “is_associated_with” relations. As all resources are already annotated with HPO phenotypes, the chatbot suggests resources to the user for other phenotypes associated with the queried phenotype.

Leveraging the DDD database allowed us to identify the concepts that co-occurred in individuals with NDDs (Figure 2). Using CAMI, we are successfully able to identify the relationship between different nodes (medical entities related to NDDs). This is possible, based on the large number of individuals in the DDD database, which makes a salient difference between the rates of co-occurrence.

**Figure 11:**
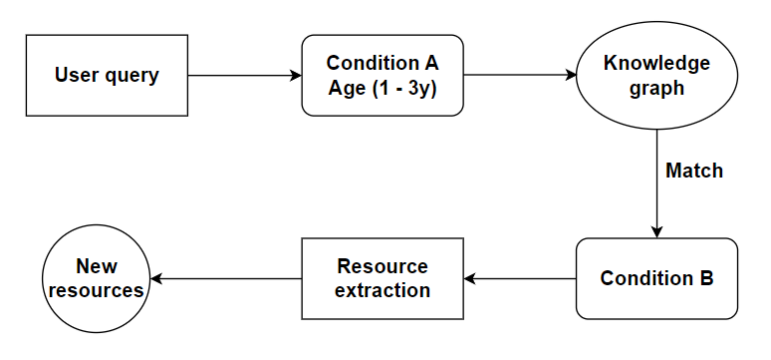
Using a knowledge graph to present the user with related conditions. The user query is subcategorized into condition and age group as parameters. These parameters are passed to the knowledge graph, and it returns the closest condition within the same or similar subgroup. The condition is passed to the resource extraction engine, and new resources are displayed to the user.

The tips or suggestions are based currently on established concepts from the coaching literature developed for families or caregivers of individuals with NDDs (Figure 11); for instance, suggesting that the user set up objectives, which is a key step and a common coaching strategy when dealing with individuals with complex conditions such as NDDs. The chatbot will provide a tip, which, if the user wishes, can be sent to them via email or SMS text message.

This allowed CAMI to provide the user with the names of conditions related to their initial query. It then offered the user the opportunity to see resources for these related entities (Figure 11).

The entities extracted from the query will be passed to the knowledge graph by calling the Neo4j database’s object instance, and the resources are matched [26]; for example, a query such as “My child has sleep issues” will be parsed and subcategorized into multiple key-value pairs. The aforementioned sample will return the result in the following format:

{HPO-DDD: [], UMLS: [Problem], EricTerm: [Sleep], AIRS: [], cb_category: [Sleep issues], location: AGE: Child, ngrams: [child, sleep, issue], relatedConditions: [], searchedCategory: [core knowledge]}

The keywords in the query, including “sleep,” “child,” and “issues,” are mapped with other medical data sets and categorized in the format shown.

### Evaluating Chatbot Outputs

#### Step 1: Internal Testing

It is important, especially for the medical domain, to assess the output of the chatbot internally with a content expert before conducting further testing. This process, known as *red teaming*, is carried out to make sure that the chatbot does not provide inappropriate results.

We started by reviewing the chatbot output with 3 caregivers of individuals with NDDs as well as the resources identified by the chatbot to assess their quality. Overall, these individuals with lived experience appreciated the resource quality; most of the suggestions were from trusted websites and did not include any advertisements or false information. The chatbot was also able to recognize, for instance, the location and age and provide different recommendations integrating this information (Table 1). 

Although query 1 and query 2 in Table 1 are similar, the top resource recommendation differs. When the age is included in the query, the system goes through a different pipeline and categorizes or ranks the resources on the basis of age as well. More specific resources will be ranked higher, and very general resources will be ranked lower. Similarly, if the location is added, then another ranking filter will be appended to the recommendations, and the list is sorted with 2 filters.

**Table 1:** Recommendations made by CAMI (Coaching Assistant for Medical and Health Information) based on user query. Top recommendations for the input query types are shown.

Query and title of web page		Reference
**“My child is suffering from ADHD [attention-deficit/hyperactivity disorder]”**		
	“Siblings of ADHD Children: Family Dynamics”	[52]
	“ADHD Behavior: Expert Discipline Skills”	[53]
	“Expert Answers to Common Questions About ADHD”	[54]
**“My child is suffering from ADHD and he is 7 years old”**		
	“Being Strength-Minded: An Introduction To Growth Mindset - Foothills Academy”	[55]
	“Baby Registry Tips: Baby Clothes | Cando Kiddo”	[56]
**“Global Developmental Delay Services in Edmonton for 10 year old kid”**		
	“Alberta Child Health Benefit | Alberta.ca”	[57]
	“Services — Qi Creative Inc.”	[58]

#### Step 2: Large-Scale Testing

Next, it is important to conduct a broader assessment of the chatbot output with a larger number of queries and evaluators. This is more challenging because patients or families have limited time. Similarly, pilot testing by clinicians is difficult to carry out.

We collaborated with undergraduate students who received training about NDDs and subsequently were asked to evaluate the resources provided by the chatbot. A total of 17 students signed up for the evaluation, but 3 (18%) dropped out, leaving 14 (82%) to evaluate the recommendations. The 14 students were divided into 3 subgroups, and each subgroup evaluated the recommendations individually for the full testing set. The evaluation sheet was divided into 4 tabs: *behavioral concerns*, *support groups or programs*, *cognitive development*, and *other topics*. Multiple user queries (from the interviews or feedback) were grouped together into each tab, and up to 50 recommendations were provided for each query. In a single subgroup, each member checked 1 query and all recommendations and answered the questions in the evaluation form (Multimedia Appendix 8). Thus, each query and its recommendations were reviewed by 3 individuals, and a majority decision (2 out of 3) was considered the final label for the evaluation.

**Figure 12:**
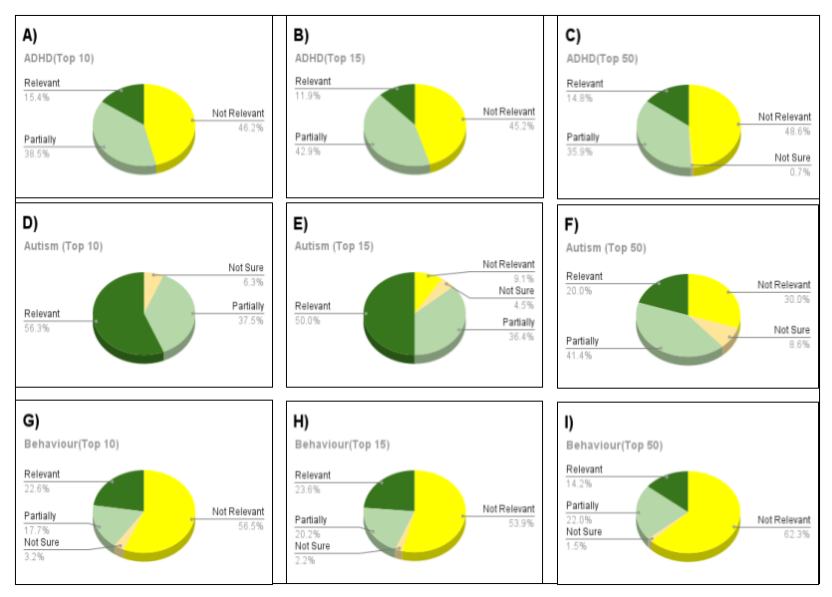
The relevance scores for top 10, top 15, and top 50 results for each topic. (A-C) Relevant resources for attention-deficit/hyperactivity disorder (ADHD). The relevant portions are almost the same for the 3 graphs, but the not relevant and partially relevant portions vary with the increase in the number of results. (D-F) Relevant resources for autism. The top 10 results show that 93.8% of the resources are relevant or partially relevant to the user query, but this percentage reduces with the increase in the number of recommendations. (G-I) Relevant resources for behavior. The performance is similar to that for ADHD, with the relevant portion increasing with the increase in the number of recommendations from top 10 to top 15 but significantly decreasing subsequently. Dark green=relevant, light green=partially relevant, yellow=not relevant, and orange=not sure.

After the evaluation, the queries were subcategorized into NDD topics such as autism, ADHD, sleep concerns, and so on, and were analyzed further (Figure 12). The analysis included the relevance scores for the top 10, top 15, and top 50 recommendations for each topic. The results varied for each topic; for ADHD, the relevance scores decreased from the top 10 recommendations to the top 15 recommendations but subsequently increased when considering the top 50 results. For autism, the relevance score gradually decreased as the considered recommendations increased, which shows that the initial recommendations are considered better and more relevant than the later recommendations. Upon analysis, we also found that the data in the database and the mapping keywords play an important role in the data ranking.

The ranking relies on multiple factors, such as location, category, Unified Medical Language System (UMLS) terms, Education Resources Information Center (ERIC) terms, type of resource required, and so on. Although we had approximately 11,000 resources that include the term *ADHD* or *attention-deficit/hyperactivity disorder*, the filters and ranking algorithms need a weighted mechanism to decide which resource to rank first. Regarding *autism*, 4169 resources in the CAMI database include the term, and CAMI performed better in terms of ranking, which shows that having more resources does not necessarily equate to better recommendations. We kept all weights at the same scale for our research, but varying weights have a better chance of improving the ranking.

## Discussion

### Summary

While chatbots are emerging as a key aspect in customer service workflow for several businesses, their use in the medical domain remains limited. Although several features of chatbots, such as 24/7 availability, accessibility from remote areas, and privacy, promise a bright future for the use of chatbots in health care, significant challenges remain; for instance, users expect chatbots to be able to process their query. This relies on NLP, and while there are extensive ontologies for general domains, an understanding of the medical domain, especially when specific to a subfield such as NDDs, remains limited. The vocabulary used by users who are lay people requires the development of synonyms for most medical terms used usually by web resources that provide either core knowledge or specialized services. We found that, in addition to using the UMLS, involving medical experts as well as individuals with lived experience could greatly improve on this. This proved to be engaging for families of individuals with NDDs because they felt involved in the development of the chatbot. We would therefore encourage developers to form, as we did, advisory groups that include individuals with lived experience.

Another important aspect to consider when developing a medical domain chatbot is the level of complexity of most medical conditions. This may be related to sex differences, age-dependent differential manifestations, or associated conditions (comorbid conditions) that may influence the clinical presentation and the needs of the user; for example, an individual with an NDD is more at risk of developing seizures. This is important information for the user to be aware of when asking about, for instance, change in behavior. However, this information may not be easily available to computer scientists developing the chatbot. Even general practitioners may not consider this information shared knowledge. Interviewing domain-specific medical experts is challenging due to access or time constraints. Therefore, we developed an alternative approach by using a large data set of information and integrating the data into a knowledge graph. This allowed us to identify coassociated conditions and provide the chatbot with knowledge of the topic.

In addition to these more conceptual aspects, several practical points need to be considered when developing a chatbot in the medical domain. Possibly the most important concerns how to manage a highly multidisciplinary team (medical, social science, and computer science professionals as well as individuals with lived experience) with very limited overlap in background knowledge. First, we noted the importance of involving potential users (caregivers) in identifying the needs of users, the features to be included, and the user interface best suited for efficient and useful mobilization of knowledge. We found that it was very important to bring together individuals who share a common interest in the topic to surmount the challenges related to differences in language and background. Indeed, we noted that an agile-inspired approach was extremely important because caregivers could better identify needs and refine approaches as they were presented with multiple iterations of the chatbot [59]. The agile approach has gained popularity because it focuses more on the potential users and the results, which enables projects to swiftly adapt to new requirements or changes as they arise.

Second, we identified several technical points that made knowledge mobilization particularly challenging for a chatbot when using automation, as opposed to manual human curation. We observed from all our user experience testing that, to be useful, the chatbot needed a wide array of resources from which to make recommendations (as was pointed out during the initial steps with the advisory group). However, we found it difficult to identify a labeled set of such resources that could be used by the chatbot. Furthermore, we noted that, for the most part, resources were labeled manually by each individual group focused on a specific health-related entity. This made it difficult for the chatbot to identify the relevant resources using a common set of vocabulary. Related to this is the difficulty for the chatbot to provide resources according to a rank most useful to users; for instance, some websites (eg, services) may have more content and therefore might include more keywords of interest than other sites with less content (which includes a list of telephone numbers for a service). At the moment, it is still unclear how simpler sites, which may be more impactful to the user, could be added.

Finally, developing a chatbot that uses a coaching approach to engage with the user and build a long-term relationship proved to be difficult. We incorporated several elements to foster engagement (customization, allowing resources to be shared, and providing expert tips), but recreating the relationship between a caregiver and a human coach remains a complex task that will require further research in mood analysis, personalization based on deeper understanding of a user’s inner mental model (which can vary significantly between users), and an awareness of the course of the disease.

### Limitations

Individuals with NDDs can present with different clinical features based on age and sex. Unfortunately, there is currently no database containing longitudinal data. In the future, user interactions, such as resource ratings of the resources combined with data on individuals with NDDs and cluster analysis approaches, could be used to recommend age-, sex-, or phenotypic profile–specific resources [60-63].

The conversation between chatbot and the user is still *scripted* to some degree, although the user can decide to branch out by either navigating to the related topics suggested by the chatbot or simply clicking on the tags presented with the resources provided for their query. In the future, we would like to expand on this and allow a question-generation system that will use the knowledge graph of entities related to NDDs to ask users questions to provide more specific recommendations [64]. Developing a question-generation system could allow parents to access required information without answering too many queries.

From a technical point of view, querying the database imposes significant load, resulting in prolonged wait times for results, which may impact the user experience. Using the correct database or adding indexing to the database queries can expedite data processing, but this will need many changes in database modeling and extraction queries [65]. In addition, regularly checking for annotation updates on websites is crucial to ensure their continued operation and content consistency. Sometimes, websites do not follow coding standards, and it is very difficult to spot abandoned or corrupted web pages and make sure that they do not show up in the suggestions. We could potentially develop a script that runs automatically regularly to find and remove pages that do not exist or have broken links. The website’s status can be confirmed by either receiving the response using the Python request module or using the Linux *ping* command [66,67]. However, the authenticity of the website needs to be reverified after the data update to ensure that false information is not disseminated.

Finally, in its current version, CAMI is only available in English, but newcomers to the country, immigrants, and people who are comfortable in a different language should be able to access the chatbot so that it can reach, and provide resources to, a larger audience [68,69].

### Future Plan

We would like to highlight the importance for our project of creating a large data set of longitudinal data to track patients’ data over a period of time using the chatbot. This will allow the system to monitor patient behavior and can provide proactive solutions or resources in the early stages itself. This type of information will also be crucial for other artificial intelligence tools aiming at predicting outcomes and assessing intervention impact.

Another key aspect of chatbots operating within the medical domain will be a precise NLP system, necessitating the creation of a lexicon of domain-specific medically relevant terms alongside layperson language that can be recognized by the language models. Along the same lines, a truly dynamic system with automatic question generation will make the chatbot smarter in terms of its conversational manner. In addition to the user asking general questions, the specificity of interactions with the chatbot will be determined by the responses or queries typed by the user. Better questions will lead to better and more specific responses and better recommendations [70-72].

## Appendix

Multimedia Appendix 1 Semistructured interview consent and questions.

Multimedia Appendix 2 Coaching Assistant for Medical/Health Information home page (top portion).

Multimedia Appendix 3 Coaching Assistant for Medical/Health Information home page (bottom portion).

Multimedia Appendix 4 Coaching Assistant for Medical/Health Information sign-up page.

Multimedia Appendix 5 Coaching Assistant for Medical/Health Information chat interface.

Multimedia Appendix 6 Coaching Assistant for Medical/Health Information resource card.

Multimedia Appendix 7 Coaching Assistant for Medical/Health Information chat radio buttons that enable users to explore related conditions.

Multimedia Appendix 8 Coaching Assistant for Medical/Health Information questions for recommendation evaluation.

## Abbreviations

ADHD: attention-deficit/hyperactivity disorder
CAMI: Coaching Assistant for Medical/Health Information
CONSORT: Consolidated Standards of Reporting Trials
DDD: Deciphering Developmental Disorders
ERIC: Education Resources Information Center
HPO: Human Phenotype Ontology
LLM: large language model
NDD: neurodevelopmental disability/difference
NLP: natural language processing
UMLS: Unified Medical Language System
