# Supplementary material for: Developing a Chatbot to Support Individuals With Neurodevelopmental Disorders: Tutorial
Source: J Med Internet Res. 2024 Jun 18;26:e50182. doi: 10.2196/50182 (PMC11220430; doi:10.2196/50182)
Supplement: Multimedia Appendix 1 [file jmir_v26i1e50182_app1.docx]

**Semistructured Interview Consent and Questions:**

Hi, . My name is , and I’m

going to be helping to guide the session today.

Before we begin, I have some information for you that

I’m going to read to make sure that I cover everything.

You probably already have a good idea of why we asked you here, but let me go over it again briefly. We’re asking people to view a demonstration of a chatbot that we’re working on so we can see if it works as intended, as well as meets the needs of the end user. The session should take about 45 minutes.

The first thing I want to make clear right away is that we’re testing the chatbot, not you. You cannot do anything wrong here.

As you watch the demonstration, I’m going to ask you as much as possible to try to **think out loud:**to say what you are looking at, what you would try to do, and what you are thinking (both positive and negative). This will be a big help to us.

Also, please do not worry that you’re going to hurt our feelings. We’re doing this to improve the app, so we need to hear your honest reactions.

If you have any questions as we go along, feel free to go ahead and ask them. I may not always be able to answer them right away, since we’re also interested in how people do when they don’t have someone sitting next to them to help. But if you still have any questions when we’re done, I will try to answer them then. If you need to take a break at any point, just let me know.

With your permission, we’re going to record the session. The recording will only be used to help us figure out ways to improve the chatbot and won’t be seen by anyone except the people working on this project. It will also help the session go faster because there won’t be a need to take as many notes.

Some of the lead programmers from the design team will be demonstrating the chatbot.

If you would, I’m going to ask you to confirm that we have your permission to record you, and that you acknowledge the recording will only be seen by the people working on the project.

Do you have any questions so far?

**General Questions:**

1. What kind of device do you usually use? (Phone, laptop, tablet)

2. What do you think the website is about?

3. On a scale of 1-10, how much would you rate the color scheme of the website?

1. Are there any colors that negatively affect your experience?

4. How comfortable are you in using the internet? (Quantify in number of hours in the week)

5. Will you trust this chatbot if you have to save some information on it?

6. Where would you click to start?

7. Did you ever used a Chatbot before?

8. Do you know what this chatbot is about? What is the purpose of CAMI?

**Go to Chat:**

1. What do you think this is about and what would be your next steps?

2. Is the introduction text enough or understandable?

3. Normally, what do you search on google or other search engines when you are looking for the information on developmental differences?

4. Do the resources make any sense to you?

5. In terms of relevancy, rate 1 to 5 for the top 5 resources

6. Do tags on the resources provide any helpful information before checking out the entire webpage?

7. Will you save the resources if you like it?

8. Will you share the resource if you like it?

9. Do you normally like to know more about the similar conditions with your searched category or you just want to look for specific information?

10. Does the image on the resource make a difference?

11. Do you think chat is intuitive?

12. Do you think the chat is too personal, not at all or just about right?

13. For the number of resources do you like that we show x number of resources or would you want to see the top 5/10?

**Future:**

1. Would you like to receive alerts on your email if we find any resource similar to what you have been suggested before?

2. Would you like it if the chatbot had a short video with explanation?

3. For sharing the resources, would you provide a number, your email, or the recipient’s email?
