# Supplementary figures and images for: Developing a Chatbot to Support Individuals With Neurodevelopmental Disorders: Tutorial
Source: J Med Internet Res. 2024 Jun 18;26:e50182. doi: 10.2196/50182 (PMC11220430; doi:10.2196/50182)

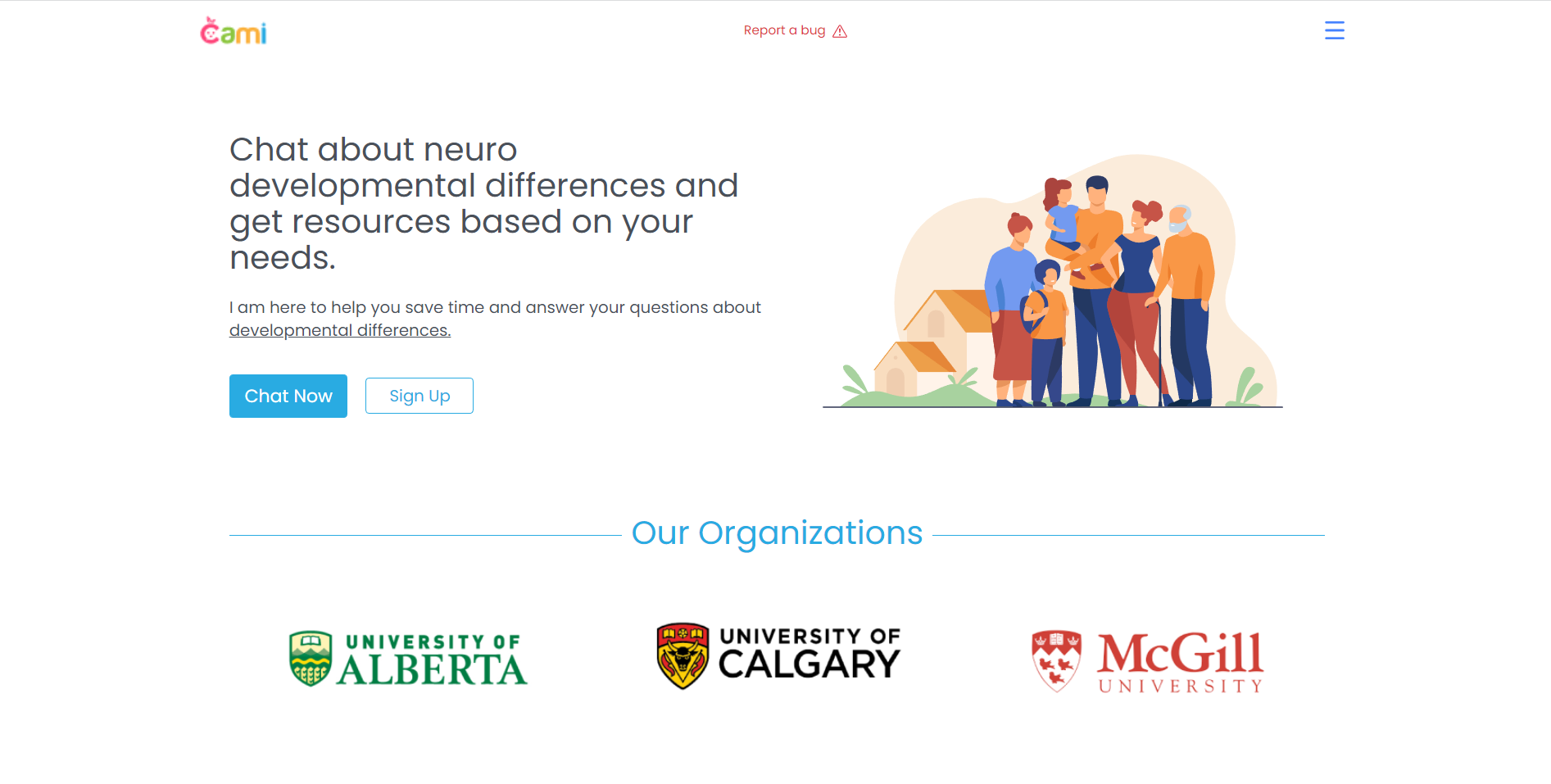

Supplement: Multimedia Appendix 2 [file jmir_v26i1e50182_app2.png]

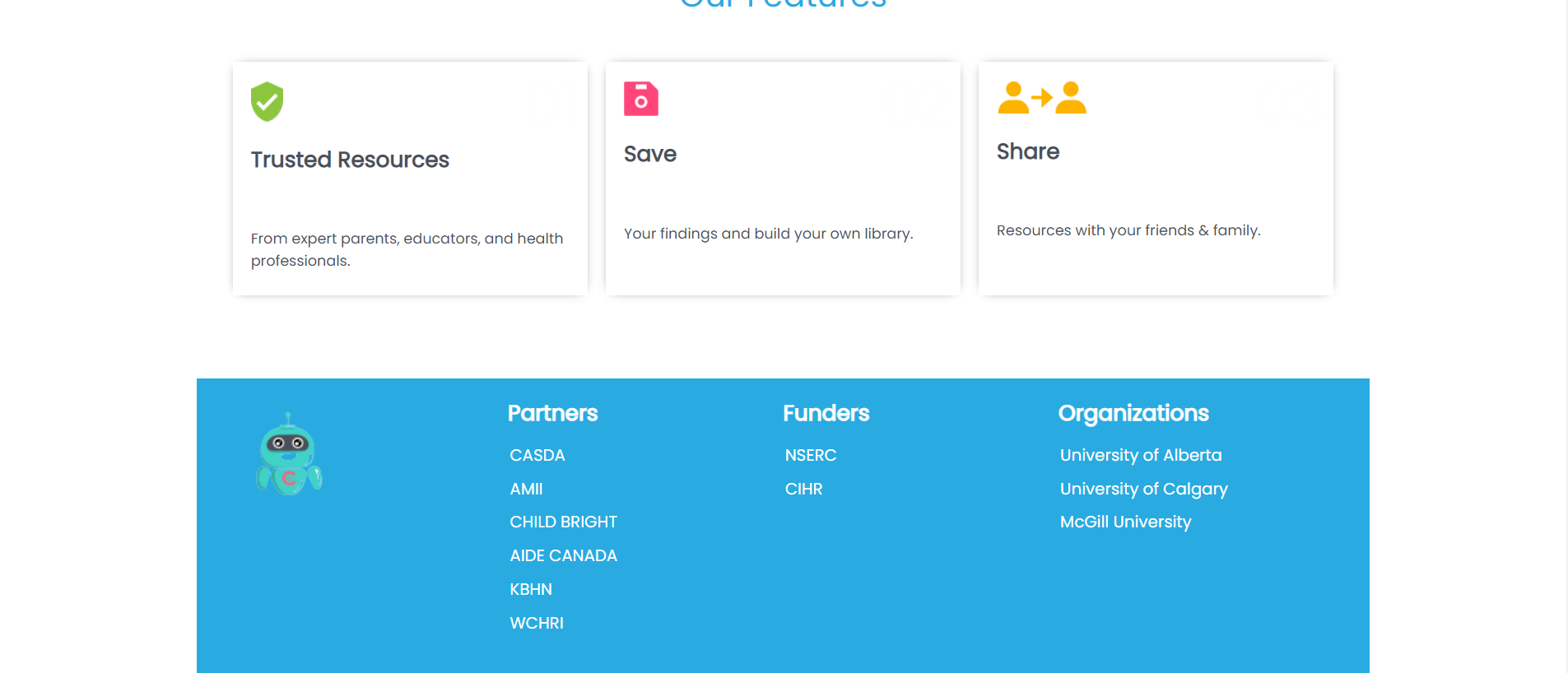

Supplement: Multimedia Appendix 3 [file jmir_v26i1e50182_app3.png]

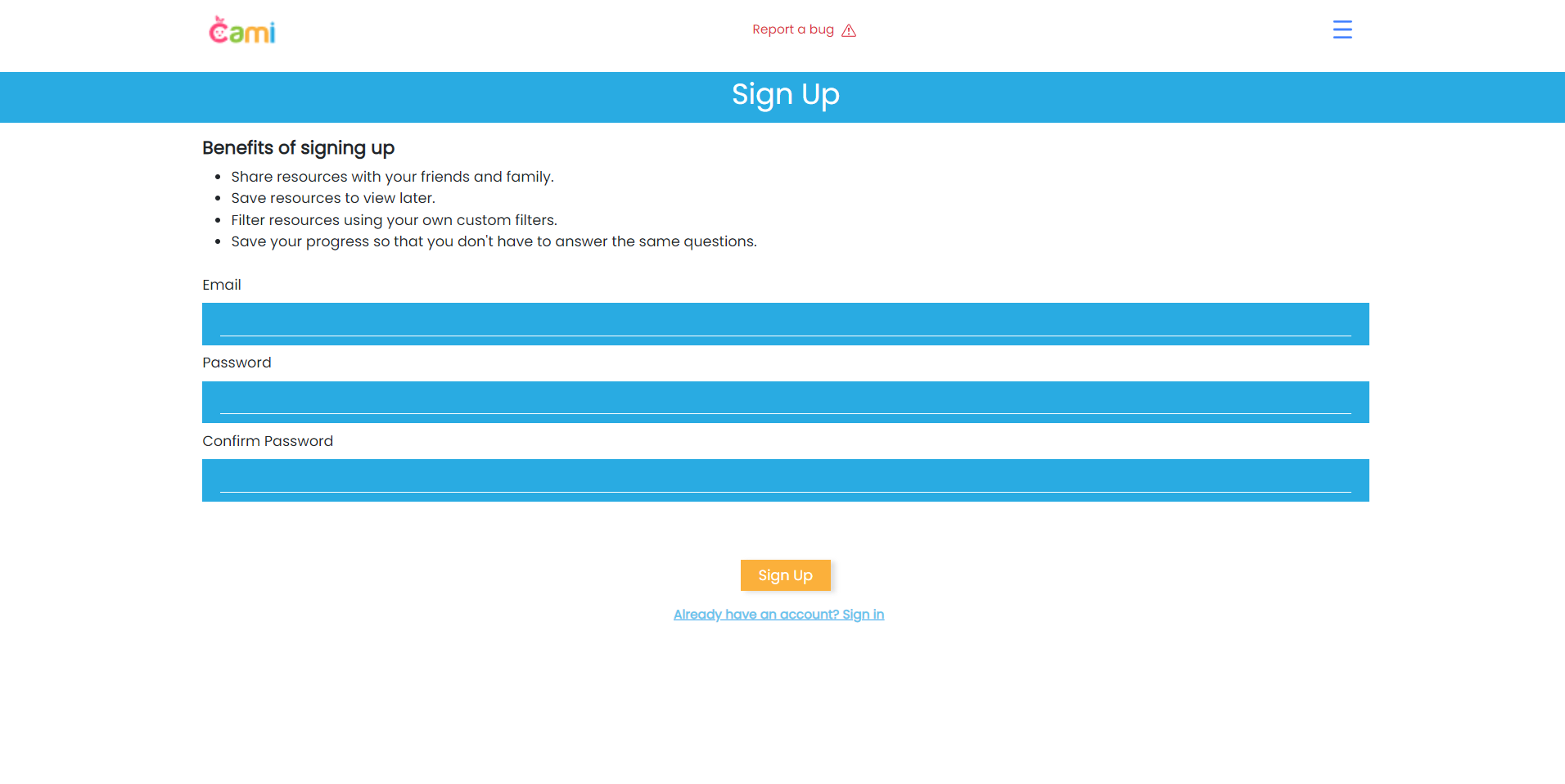

Supplement: Multimedia Appendix 4 [file jmir_v26i1e50182_app4.png]

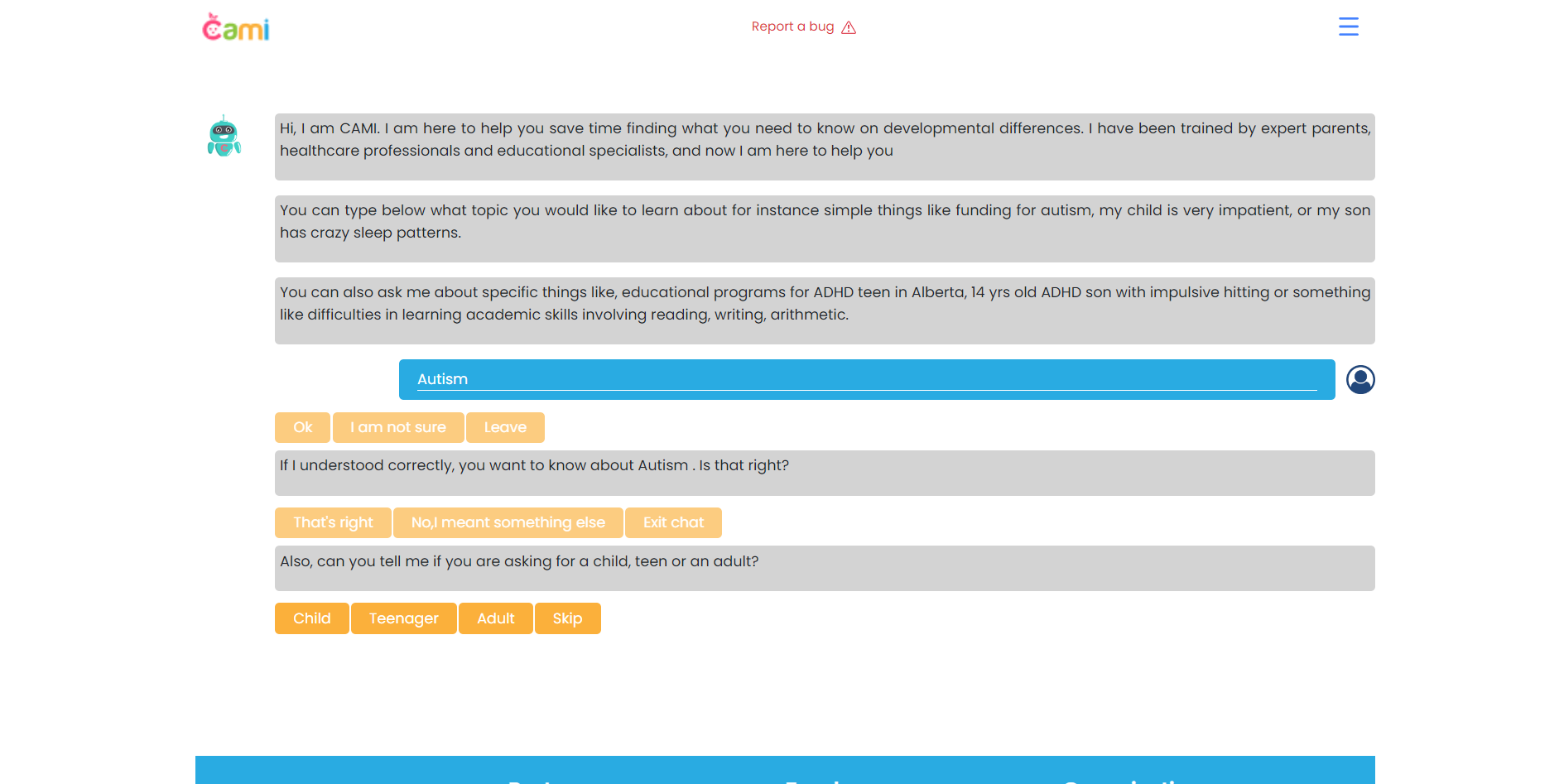

Supplement: Multimedia Appendix 5 [file jmir_v26i1e50182_app5.png]

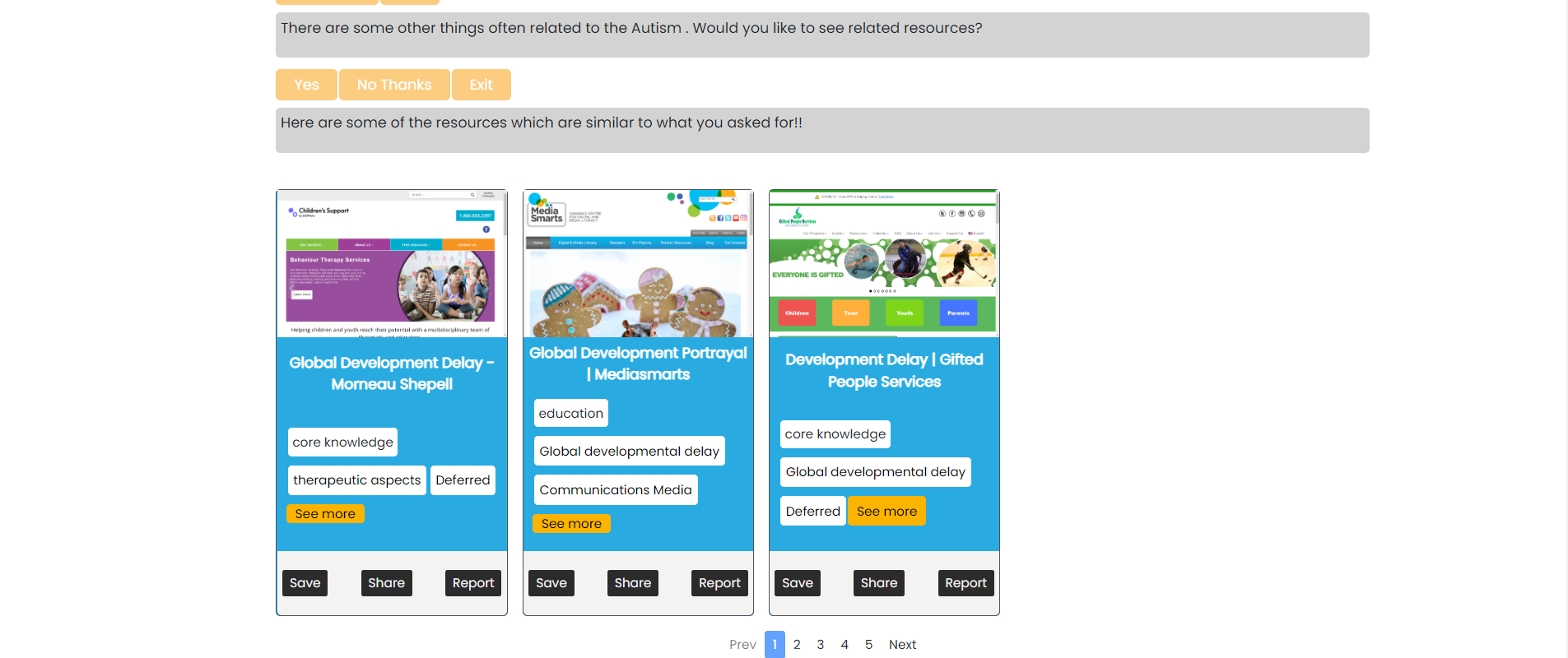

Supplement: Multimedia Appendix 6 [file jmir_v26i1e50182_app6.png]

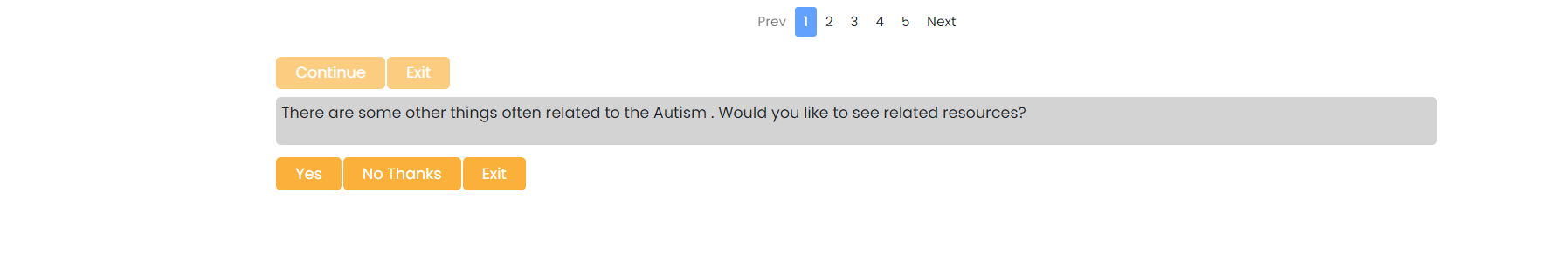

Supplement: Multimedia Appendix 7 [file jmir_v26i1e50182_app7.png]
