# Supplementary material for: Developing a Chatbot to Support Individuals With Neurodevelopmental Disorders: Tutorial
Source: J Med Internet Res. 2024 Jun 18;26:e50182. doi: 10.2196/50182 (PMC11220430; doi:10.2196/50182)
Supplement: Multimedia Appendix 8 [file jmir_v26i1e50182_app8.docx]

**Questions for recommendation evaluation:**

1. Enter the Query or User Input you are analyzing
2. Enter the URL of the recommended Resource
3. How much this webpage is relevant to the Input Query?
   **Options**: Relevant; Partially Relevant; Not Relevant;Not sure
4. Do you think the content of this webpage is Authentic or accurate?
   **Options**: Yes, the information in the webpage is accurate;No, the information is wrong or inaccurate; Not Sure
5. Is this webpage specific for a particular location?
   **Options**: Yes; No; Not Sure
6. Is the ranking presented on the Google form is accurate? If not, what would you rank the webpage?
